# Translation of tissue-based artificial intelligence into clinical practice: from discovery to adoption

**DOI:** 10.1038/s41388-023-02857-6

**Published:** 2023-10-24

**Authors:** Alice Geaney, Paul O’Reilly, Perry Maxwell, Jacqueline A. James, Darragh McArt, Manuel Salto-Tellez

**Affiliations:** 1 Sonraí Analytics, Whitla Medical Building, 97 Lisburn Rd, Belfast, BT9 7BL UK; 2 Precision Medicine Centre of Excellence, The Patrick G Johnston Centre for Cancer Research, Queen’s University Belfast, Health Science Building; 97 Lisburn Road, Belfast, BT9 7BL UK; 3 Northern Ireland Biobank, The Patrick G Johnston Centre for Cancer Research, Queen’s University Belfast, Belfast, BT9 7AE UK; 4 Integrated Pathology Unit, Division of Molecular Pathology, The Institute of Cancer Research London, 15 Cotswold Rd, Sutton, SM2 5NG UK

**Keywords:** Biological techniques, Biomarkers

## Abstract

Digital pathology (DP), or the digitization of pathology images, has transformed oncology research and cancer diagnostics. The application of artificial intelligence (AI) and other forms of machine learning (ML) to these images allows for better interpretation of morphology, improved quantitation of biomarkers, introduction of novel concepts to discovery and diagnostics (such as spatial distribution of cellular elements), and the promise of a new paradigm of cancer biomarkers. The application of AI to tissue analysis can take several conceptual approaches, within the domains of language modelling and image analysis, such as Deep Learning Convolutional Neural Networks, Multiple Instance Learning approaches, or the modelling of risk scores and their application to ML. The use of different approaches solves different problems within pathology workflows, including assistive applications for the detection and grading of tumours, quantification of biomarkers, and the delivery of established and new image-based biomarkers for treatment prediction and prognostic purposes. All these AI formats, applied to digital tissue images, are also beginning to transform our approach to clinical trials. In parallel, the novelty of DP/AI devices and the related computational science pipeline introduces new requirements for manufacturers to build into their design, development, regulatory and post-market processes, which may need to be taken into account when using AI applied to tissues in cancer discovery. Finally, DP/AI represents challenge to the way we accredit new diagnostic tools with clinical applicability, the understanding of which will allow cancer patients to have access to a new generation of complex biomarkers.

## Tissue-based AI—definition and scope

Morphological analysis has been the cornerstone of cancer tissue discovery and diagnosis since pathology became a clinical discipline at the beginning of the twentieth century [1]. To do so, pathologists apply a process of analysis and interpretation of the histological phenotype, a process that requires a mixture of innate capacity and significant pattern recognition training coupled with medical knowledge. Traditionally, these images have been prepared in glass slides and viewed by the pathologist through microscopes. Digital pathology (DP) is the acquisition, management, sharing and interpretation of pathology information—including slides and data—in a digital environment; digital images are acquired *via* scanning of glass images, and visualized on computer screens [2]. The digitization of the diagnostic services not only brings substantial operational advantages [2]; it is also an enabler for the application of in silico algorithms, created by machine learning (ML) or artificial intelligence (AI) analysis, to improve patients’ diagnosis or therapeutic decision-making. Although ML and AI are often used interchangeably, they are generally considered to be different—ML refers to the adaptive creation of in silico models of the problem domain from data and stimuli obtained from that domain to solve a particular problem. AI is a subset of this, but whereas ML will often make use of specified features and use these to train its model of the domain, AI attempts to mimic the more general process of intelligence but applied within the specific problem domain. This involves some form of ‘training’ as per ML, but typically the assumptions and constraints on the model are fewer, with little a priori knowledge being assumed. Hence the AI will attempt to learn the optimal features required for a particular task, as well as the mapping of those features to optimally solve a particular problem. The application of AI to DP images, which is the core to this article, will be referred to as DP/AI henceforth.

## The evolution of medical imaging from image analysis and machine learning to AI

Although image analysis (IA), ML and AI are often used interchangeably, it is important to expand on the differences between them as highlighted above, from a conceptual and also historical perspectives.

IA is the set of longstanding methods of processing digital images—usually a single-channel format such as grayscale images, or a multi-channel format such as Red-Green-Blue (RGB) or Hue-Saturation-Value (HSV), which encodes the detail of images (and colours in the case of multi-channel images). This processing aims to extract features such as edges, textures and colour variation from the pixel values of the images, which are then used for visualization purposes or further used in downstream tasks such as classification or segmentation.

The term machine learning (ML) is usually applied to any of a broad set of methods whereby computers can use data, or features derived from those data, to ‘learn’ patterns and correlations within the data, as applied to tasks such as classification or segmentation. This may be as simple as linear regression, or encompass more sophisticated approaches such as Support Vector Machines or Random Forest Classifiers. In the past, ML was typically paired with IA-derived features to solve medical imaging problems. One difficulty with this approach is the need to select or aggregate the appropriate features for input into the ML algorithm, which was often done heuristically and not optimally for the end-to-end task of processing the image pixels and performing the goal.

With AI, that ‘feature engineering’ step is typically avoided. Although AI encompasses IA and ML in some ways (images are processed, and patterns are learnet from the data), the features, as well as the correlations and patterns, are learnt from the data alone—hence the important image features are optimised for the task at hand. Whilst this typically requires more data to train a generalizable model than traditional IA/ML solutions, across the domain of image processing AI has been found to be much more powerful for most tasks.

## What can tissue AI offer today?

Tissue pathology is based on the correlation of disease entities (and sometimes physiopathological processes) with specific histologic and cytologic appearances. The link between appearances and disease is, translated into a diagnostic opinion, the backbone of traditional pathology, and arguably one of the most common diagnostic strategies routinely used in modern medicine. To achieve this goal, the training of tissue pathologists is intuitive, poorly explained, and epistemologically is probably linked to gestaltism [3]. This has been a successful approach in modern medicine; it is affordable, reproducible to a certain extent, and widely applied in routine diagnostics.

**Fig. 1:**
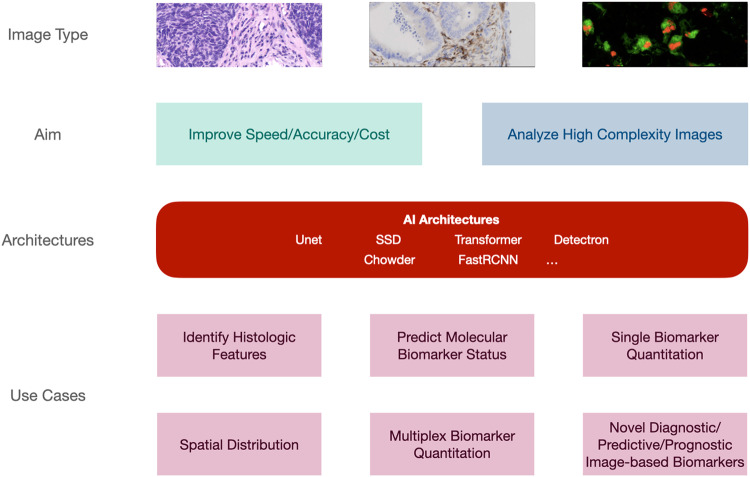
AI Applicability: levels of AI applicability in the diagnostic setting—an illustration of the applicability of AI architectures to assist achieving the aims of the pathologist for a number of use cases. Unet U-shaped architecture, SSD single-shot detector, FastRCNN Fast Region – Convolutional Neural Network.

More than 10 years ago, Beck et al. [4] hypothesized that a systematic interrogation of morphological features by ML could increase the number of phenotypic characteristics with clinical significance, improving accuracy and reproducibility in diagnostics. Since then, a significant body of knowledge has been created to apply ML and AI to digital scans of tissue-based images. This is percolating the field at many levels (see Fig. 1). Indeed, DP/AI can today complement traditional pathology methods by improving diagnostic speed and accuracy, with efficiencies in diagnostic delivery and cost; identifying histological features [5], key to cancer diagnosis or patient stratification, with an accuracy similar or superior to traditional pathologists [6]; predicting the status of molecular biomarkers in basic hematoxylin-eosin slides close to nucleic acid-type gold-standards [7]; quantitating clinical biomarkers, tested today with standard chromogenic hybridization approaches, with tools currently utilized as “assistance” to routine reporting [8]; quantitating clinical biomarkers in technology platforms untested in the clinical setting to date [9]. In addition, DP/AI has the potential of providing quantitative, reproducible analysis of tissue-based tests holding significant complexity, opening a new generation of diagnostics. This can happen in the context of analyzing novel, explainable approaches to cancer ecology and spatial distribution with clinical significance [10], or based on AI’s ability to mine subvisual image features, thus bringing us to the interesting and controversial space of “unexplained AI” in diagnostic imaging [11]. Indeed, one of the benefits of pursuing regulatory approval of such novel AI approaches (as with any other diagnostic approach) is the need for advanced or novel visualizations to contextualize decisions made by the AI.

To date, most DP/AI tools approved by regulatory agencies are designed as a means of “assisting” the pathologist in the diagnostic process, rather than “substituting” the pathologist in this endeavour. Two important reasons are behind this approach: firstly, the reluctance of the diagnostic community in adopting the latter; but secondly and more importantly, the perception that the available tools still require a level of phenotypic supervision by a practicing pathologist before their result can be applied in a clinical context. This is epitomized by the shortcomings and limitations reviewed elsewhere [5] and that, in summary, include: lack of access to large well-annotated data sets in the development phase; context switching between clinical workflows; algorithms not fully integrated in the diagnostic pathways because they are slow to run or require individual configuration; lack of properly defined protocols for training evaluation; overall lack of proper validation, or health economics studies supporting their utilization against traditional gold-standards; absence of clear evidence of added value in everyday clinical decision-making; and the possibility of false negative results or missed diagnosis. The latter has been elegantly illustrated by Echle et al. [12]. Deep learning systems increase their performance by adding cases to train the algorithms, up to a point in which they reach a “performance plateau”. The final result often falls short of clinical applicability. All of these shortcomings and limitations should be addressed when taking a potential new tool through the regulatory clinical flow (see below).

## Types of AI applicable to tissue pathology

AI architectures have been developed for a number of different tasks and in a number of different domains – principally in the areas of language modelling and image analysis. Before image analysis, natural language processing (NLP) is being applied to pathology reports and other medical records [13], with application to classification and the extraction of consistent, and pertinent, information from variable sources.

Given its visual nature, it is unsurprising that AI has been applied with some success to the analysis of pathology images. The increased use of digital pathology scanners to produce high resolution Whole Slide Images (WSI) has enabled researchers to build up cohorts of images across multiple disease indications, tissue types and stains [14, 15]. These can be analyzed by a variety of AI-based techniques, predominately Deep Learning Convolutional Neural Networks (CNN), which have been found effective for analysis of images across multiple domains [5]. The size of WSI compared to the images from those domains is a challenge, but this is typically addressed by dividing the slide images into tiles or patches, which are more easily used in AI pipelines.

CNN-based AI may be used for a variety of different tasks on its input images, namely classification, segmentation and regression. Classification is the assigning of a single label to an image and may be a binary classification (such as tumour presence) or multiclass (tissue type and tumour grade and tumour stage, for instance). Segmentation provides a more detailed classification of pixels, areas or objects within an image, and may be semantic (i.e. pixel-wise) or instance-based (i.e. segmenting distinct areas or objects). The regression task predicts a continuous variable based on the contents of the image. Given these fundamental capabilities, which particular architecture or approach is employed depends on the requirements of the pathology user. The following details common tasks in the pathology domain to which AI has been applied.

### Tumour detection

**Fig. 2:**
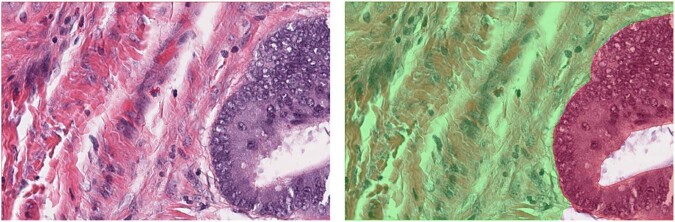
Segmentation of Tumour in H&E Images. H&E image showing tumour and extra-tumoural stroma (left) and ground truth segmentation of the compartments (right) tumour in red and stroma in green.

The most common use of AI is the detection of tumour in haematoxylin and eosin (H&E) images. This is typically based on classification or semantic segmentation algorithms, detecting areas of the WSI (usually in H&E-stained slides) which are suspect for tumour. Such algorithms may also aim to provide a classification of the different compartments within the tissue, or subclassify the tumour further according to grade or other diagnostic criteria. Figure 2 shows an example of the ground truth segmentation of tumour and extra-tumoural stroma in colorectal cancer (CRC). Such approaches have been successfully applied to prostate [16] and breast cancer [17], for example. The application of tumour detection is obviously directly applicable to diagnostic use, and potentially can bring advantages in consistency and workflow efficiency. However, outside of that use case, the task of tumour identification is a precursor to many other analyses, from identification of regions for macrodissection of tissue for downstream molecular testing, to generation of Regions of Interest (ROI) for immunohistochemistry (IHC) scoring [5].

### Case classification

Multiple Instance Learning approaches have been utilized to train AI algorithms which allow the prediction of a slide-level classification (such as cancer type) from a WSI directly. Indeed, some of these products provide a slide-level indication of cancer status, and have been widely validated [18]. Fremond et al. [19] have detailed an approach for such slide-level prediction of molecular classification in endometrial cancer with validation results using multiple cohorts. In the area of colorectal cancer, Echle et al. [20] have shown that AI approaches can be used to predict microsatellite instability (MSI) status across multiple validation cohorts. The ability of AI approaches to predict molecular status from H&E images promises efficiencies over alternative ‘wet lab’ testing or IHC scoring, and since they are based on the scanned image of a single prepared slide, may be especially useful in cases where there is little tissue available for downstream testing.

### Quantification

A less common, though growing, area of interest is the use of AI to quantify characteristics of the tissue at a cellular level. This applies to both H&E and IHC-stained tissue, and uses semantic/instance segmentation or object detection techniques to identify nuclei and/or cells matching a particular characteristic (those which are neoplastic, or those which are positively-stained, for example) within the tissue. This subsequently allows for these detections to be quantified in a consistent and repeatable manner. In the realm of spatial analysis, accurate detection of nuclei and cells can allow such relationships to be analyzed. As an example, Sarker et al. have applied deep learning segmentation algorithms to the quantification of ICOS expression in IHC-stained slides of colorectal cancer [21]. Using similar approaches, Foersch et al. have shown that the use of Deep Learning-based quantification algorithms can be applied to multiple IHC-stained tissue sections and applied prognostically and predictively [22].

### Regression

The outputs of quantification algorithms as detailed above have been used as input to regression models for prediction and survival. However, ML and AI models can also be used to produce such outputs directly. Using such techniques as attention gating, the relationship between image features extracted by Deep Learning and an output such as risk score can be modelled. Details of such an approach are given by Courtiol et al. [23], with application to predicting survival of patients with malignant mesothelioma. Such an approach has also been applied in the clinical product domain, which produces a patient recurrence risk score based on H&E images of breast cancer [24] Other publications have shown the utility of such approaches in bladder cancer [25, 26] and glioblastoma [27]. Given that these are completely novel ‘digital assays’ compared to replication of existing diagnostic tests, it is not sufficient to validate such tests with a retrospective non-inferiority or concordance study. The establishment of such tools as novel diagnostic ‘digital assays’ will require more comprehensive study design and follow-up to ensure their safety and utility within clinical pathways.

### Clinically approved tools

**Fig. 3:**
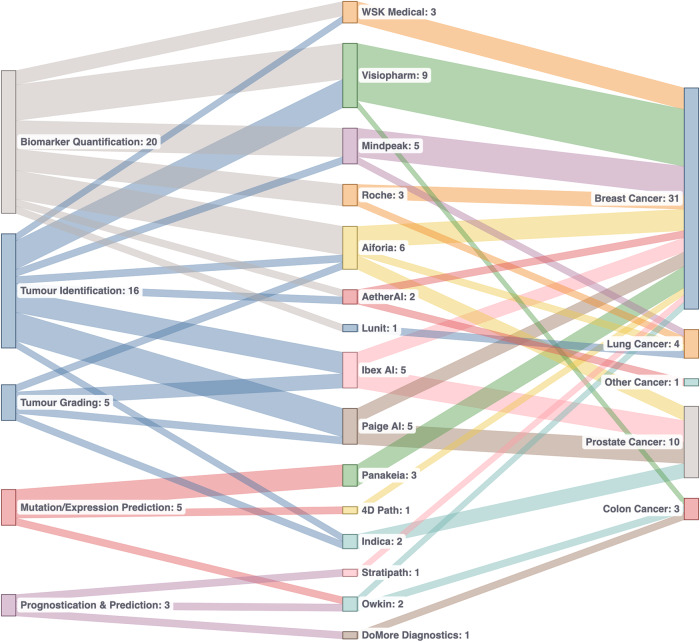
Overview of currently-approved CE-IVD products with manufacturer, task and indication (last checked on 09/2023). It is notable that the majority of approved applications are for tumour identification and biomarker quantification in prostate and breast cancer. In addition to the high rates of prevalence of these cancers, the tasks of tumour identification and biomarker quantification are time-consuming and difficult, and AI can improve efficiency and consistency of these in the clinic.

There are currently a number of tools which have approval from regulatory bodies in the EU and US which use AI to address particular challenges, across a number of different indications, and for a number of use cases (as shown in Fig. 1, and discussed above). Figure 3 summarizes the current state of play in regulated AI pathology applications. The majority of applications are intended for use for breast and prostate cancer, and the most common use cases are assistive applications for tumour identification and biomarker quantification. This is driven by the prevalence and scope for efficiency and/or consistency improvements using an algorithmic approach. However, as described above, there are applications in a number of other indications and for other use cases, such as molecular status inference and for prognostic/predictive purposes. These latter use cases are attracting a lot of research interest and the expansion of such solutions is likely to increase in the future.

## The tissue AI tool validation: similarities and differences from biomarker testing in routine diagnostics

The first tests aiming to predict eligibility for specific therapeutic interventions were introduced more than 30 years ago [28] and, since then, a battery of tests (mostly related to tissue hybridization and nucleic acid-based testing) have been successfully applied to cancer patient management. As any other medical device, these tests have an inherent degree of risk associated with their use, this holds for both traditional and digital pathology in vitro diagnostics (IVD) devices. An essential aspect of device design is assessing the performance and safety of the device as part of design verification and validation, usability testing and subsequent performance evaluation activities to ensure the device performs as expected on newly tested samples.

The performance evaluation testing requirements are similar no matter the type of IVD device. The requirements of International Medical Device Regulators Forum (IMDRF), In Vitro Diagnostics Regulation (IVDR) and the various other regulatory requirements associated with performance evaluation provide clear guidance about how the performance of any IVD device should be assessed. In brief, the steps are as follows:

1. Determine the scientific validity of the analyte with the clinical condition or physiological state.
2. Carry out relevant analytical performance testing to determine if the device can correctly detect or measure the selected analyte.
3. Where necessary, conduct clinical performance studies to assess the ability of the device to yield results that are correlated with the intended clinical condition or physiological or pathological process or state, when being tested on the target patient population, by the intended user of the device, over the expected lifetime of the device.

The level of performance evaluation testing is dependent on the novelty of the device in terms of novelty of the analyte, technology, patient population when associated with the analyte and/or technology, new application of an established technology, or a new intended use which is not established or standardized.

The appropriateness of clinical evidence from a quality and quantity perspective needs to be determined early in the design phase for novel biomarkers and novel technologies to confirm the device is safe, and achieves its intended clinical benefit. When determining the quality of the information gathered, the reviewer should consider: how appropriate the study type and design to meet the research objectives; the suitability of the dataset and was it state of the art; and the appropriateness of the statistical approach to reach a valid conclusion.

When determining the quantity of information to gather, the reviewer should consider, for example: the suitability of the data to support the intended use, indications, contraindications, target groups, intended user, clinical claims, residual risks and intended user environment; a determination if the clinical risks and analytical/clinical performance have been investigated; and a determination if the relevant characteristics (e.g. cross-reactivity) have been considered to support the performance of the device.

From the very beginning of test validation, more than 30 years ago, it was clear that different approaches would require different validation pipelines and frameworks [29]. A question that is paramount to DP/AI- based biomarker discovery, is that design verification and validation of such tests differ greatly between physical devices with reagents, hardware and consumable equipment versus software devices which use images or other data inputs for analysis. Design verification of physical devices can be performed on individual components of the overall kit/system as the component is locked down, and validation can be performed in a similar manner depending on the relationship of the kit/system components.

Good ML practices for AI model development includes a stage for model validation, this is an involved stage where tests using various models and test criteria are used.

The model validation planning should include definition of the target quality metrics of the model and definition of testing activities for the model and specific features used by the model to make decisions. A strategy should be established and executed to test the model and develop explainability which can be provided to users in accompanying literature.

Analysis of the results of the testing is paramount to assess the performance of the models alongside the respective datasets to identify any potential safety or performance risks, and enable subsequent additional testing.

Software only device design is more agile as the discovery phase of development can enable manufacturers to informally test aspects of the device as development is ongoing. However, challenges arise as the formal device verification activities are generally performed immediately before formal validation activities, as the device must be locked down to ensure it is being tested as a complete package. Ensuring testing is being performed during software development is key to avoiding unexpected device failures during formal testing which, depending on the nature of the failure, could mean the device does not meet the user needs or the benefits of the device do not outweigh the risks (both safety and security).

Another difference in AI and traditional biomarker testing, is how the output of the testing is communicated to the user. Traditional devices can present information on the sensitivity and specificity for example, however the output of AI devices should also include information on how the algorithm came to its decision on a case by case basis, this information should be gleaned from development activities and tested during design verification and validation and clinical performance testing. The user’s understanding of the explainability information should be assessed, and included as part of the overall clinical benefit assessment, as user misinterpretation of the results or overtrust pose real risk to patients.

## Tissue AI: clinical utility (tissue AI and clinical trials)

From target discovery to the development of a companion diagnostic algorithm, DP/AI applications are key in the process of biomarker development parallel to the development of new drugs [30]. DP itself is a facilitator of remote and multi-viewer access to study materials, from target discovery to clinical trial sample analysis. The quantitative approach facilitated by ML and AI can support adequate biomarker analysis at the stages of drug discovery, preclinical studies and clinical development. Together, DP/AI should provide more accurate and reproducible new tests in the area of personalized medicine. At the same time that these advantages are recognized, others have highlighted the basic requirements that would need to be addressed, and agreed upon by the clinical trial study designers, before any form of image analysis is adopted. These include: standardization of digital pathology laboratory procedures; adequate funding allocation and governance framework; definition of performance criteria of image analysis approaches upfront; engagement with regulatory bodies; and a proper framework for deployment of DP/AI tools subsequently [31].

**Fig. 4:**
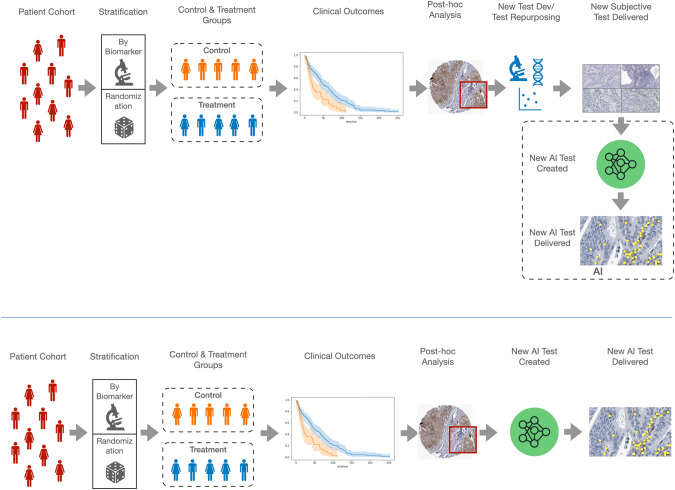
Current and future model for AI integration in clinical trials. The current process of AI-facilitated biomarker testing it the clinical trial context (as a surrogate on an “interpretative” test developed beforehand, top panel; versus the proposed "de novo" development of an AI-based test, bottom panel.

Incorporating DP/AI into clinical trials can have a transformational effect in the true clinical value of quantitative biomarkers but, as indicated elsewhere, may require a change of paradigm in the way clinical trials are designed [32]. This is depicted in Fig. 4 (top panel) presents the current model of predictive biomarker generation in a regular clinical trial design; here, the DP/AI test performance is dictated by a biomarker test design in a traditional, semi-quantitative manner at most. Figure 4 (bottom panel) presents the ideal scenario, where a direct use of clinical trial materials allows a DP/AI test, with the accuracy (and, most importantly, reproducibility) that a routine test would require. The delivery if such test would require tissue pathology diagnostic services fully digitized and with the image management systems able to apply such new algorithms routinely.

## Tissue AI: road to clinical accreditation

As with all medical devices the regulatory landscape is changing, with the flurry of new guidance documents and complexity of navigating regulatory requirements in different jurisdictions—medical device manufactures need to ensure they are keeping abreast with changes in requirements and feeding those into their quality systems and design activities in a timely manner. None more so than those manufacturers designing AI/ML medical devices.

Internationally, the IMDRF/Artificial Intelligence Medical Devices (AIMD) Working Group, IEEE P2801 Artificial Intelligence Medical Device Working Group and ISO/IEC JTC1/SC42 Artificial Intelligence Committee are establishing AI/ML guidance and standards which, in the authors opinions, will become an expectation of regulators over time. These include ISO/IEC TR 24027:2021, IMDRF/AIMD WG/N67, ISO/IEC TR 29119-11, BS 34971/AAMI CR 34971 with more to come.

In Europe with IVDR/MDR there are no harmonized standards specifically for AL/ML devices, however, Notified Bodies are participating in white papers discussions [33–35]. Johner et al. have provided an extremely useful source of information [36], combines requirements into a checklist for manufactures and regulatory authorities to use as part of establishing processes and creating design documentation. The AI Act in Europe is causing concerns about conflicting requirements, misalignment of risk classifications and the need to have conformity assessed by two different bodies. In the USA, the FDA have issued the ‘Artificial Intelligence and Machine Learning (AI/ML) Software as a Medical Device Action Plan’ [37], within which they confirm an intention to ‘encourage harmonization of Good Machine Learning Practice development’ and are releasing guidance documents. Interestingly, the FDA acknowledges that ‘medical device regulation was not designed for adaptive artificial intelligence and machine learning technologies’ [33–35] and they will drive change to the legislation.

In the UK, the MHRA guidance on Software and AI as a Medical Device Change Programme - Roadmap [38] details MHRA plans and requirements, and the AI Standards Hub is a useful source of information, discussion forums and training.

Detailed information is available on the regulators’ websites about their review process(es), so the following discussion will focus on the road to clinical accreditation, which can be broken down into 4 main areas: Quality System > Design and Development > Performance Evaluation > Regulatory Approval.

### Quality system

To access major markets, establishing an ISO 13485:2016 compliant QMS is widely accepted. EN 62304 and EN 82304 are internationally recognised standards for software development life-cycle (SDLC) and health software, and processes built from these can be easily adapted to include specific AI/ML requirements for data selection and management, feature extraction, machine learning operations, model versioning, model validation and error analysis. In addition to SDLC processes, risk (both safety and security) management, usability, labelling, vigilance, installation, maintenance and post-market performance monitoring processes will require update to include AI/ML requirements.

**Fig. 5:**
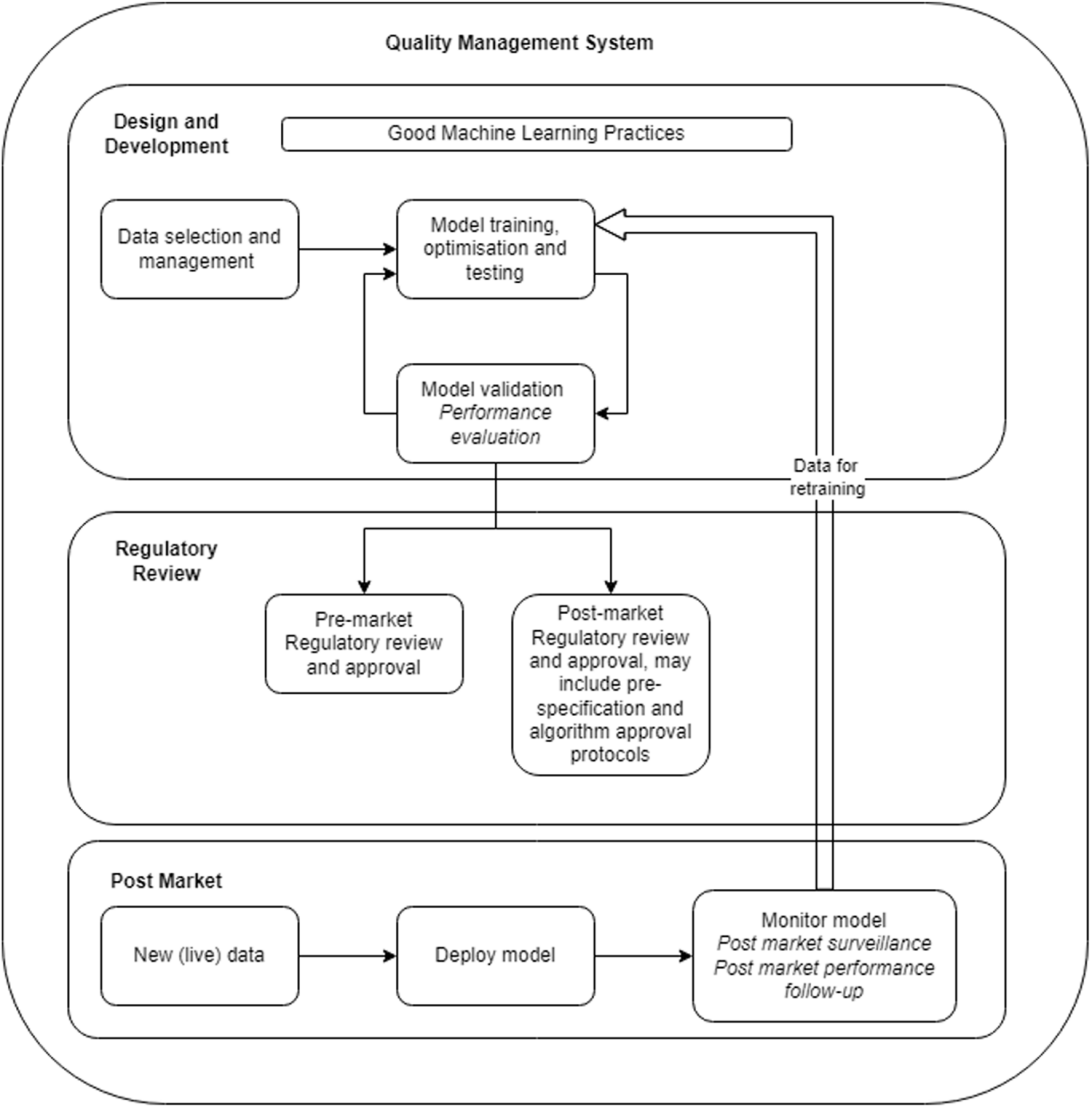
AI/ML medical device workflow road to clinical accreditation, adapted from [45]. This figure intends to present the relationship between the pre-market, regulatory approval and post-market stages of an AI/ML device.

Care should be taken when building QMS processes to ensure processes have been put in place which meet the requirements of the target jurisdiction. Forward planning is also needed to ensure future market requirements are built-in or processes are adaptable enough to enable update without adding extra burden to the business and design teams. Figure 5 aims to highlight the AI/ML aspects which need to be taken into account as part of QMS process required under ISO 13485:2016. Regulatory activities should focus on AI/ML specific legislation to guarantee sufficient information is available for external review and appropriate quality processes are in place to monitor model performance when devices are in clinical use to ensure timely action can be taken to ensure ongoing safety and performance.

### Design and development

The design and development requirements for AI/ML medical devices are evolving and manufacturers are responsible for ensuring all appropriate process and product requirements have been identified. Table 1 intends to briefly outline the known process requirements using the ‘Good Machine Learning Practices for Medical Device Development: Guiding Principles’ created by the FDA, MHRA and Health Canada, from identifying the design team, definition of the intended purpose and the design requirements, establishing data management and machine learning operations for the model and risk management activities specific to AI/ML device types, to define the monitoring and maintenance process of such devices. Note this is not an exhaustive list.

**Table 1:** The table below uses some (less self-explanatory parts of) ‘Good Machine Learning Practice for Medical Device Development: Guiding Principles’ created by the FDA, MHRA and Health Canada, to discuss AI design requirements.

**Design and development**	
Design team	Ensure the design team includes technical, clinical and regulatory specialists, with relevant experience. Having a multidisciplinary team involved from the beginning helps ensure all aspects are considered early in development. Clinical key opinion leaders are crucial to understanding the intended purpose of the device in the currently accepted workflow and supporting risk assessments during development and final benefit/risk acceptance.
Intended purpose, state of the art and target performance	Clearly define the intended purpose, ground truth, target performance, and model hyper parameters. This information will be used to determine the state of the art for the device and the statistical rational for the training, testing and validation datasets.
Data management and machine learning operations	Establish data management plans for selection and handling of the data sets used as part of the training, testing and validation. Include requirements for statistical rationale for the size of the data sets used, quality acceptance criteria, details of how out of specification samples are handled, tools used as part of the data preparation and analysis, roles and responsibilities for data preparation and approval (with necessary independence), metadata that is required for samples and is to be collected as part of the data preparation activities, number of samples and statistical rational, description of the machine learning pipeline, how is version control maintained, details of error analysis.
Risk management	Risk assessments need to include risks associated with the use of the device in the clinical workflow, use error, security risks and risks associated with AI/ML as a technology, for example bias built into the model by an inadequate sample cohort. BS 34971/AAMI CR 34971 is a useful source to understand the application of ISO 14971 to Artificial Intelligence and Machine Learning Ensure cybersecurity risk assessments are initiated early in the development process and are updated as the development evolves. The ensures vulnerabilities in the AI/ML device and/or connected platform or environments are considered. There are many guidances available from MDCG 2019-16 [42], FDA Cybersecurity in Medical Devices: Quality System Considerations and Content of Pre-market Submissions (Apr-2022) [43].
Design requirements	Taking time to establish appropriate design architecture and ensure design teams are communicating to ensure all data transfer requirements between and within devices is clearly identified will help avoid unexpected failures in verification. Review harmonized/consensus standards to identify any device specific requirements.
Human-AI team	Use scenarios and the human-AI team should be considered when establishing user interfaces and workflow steps, engagement of key user profiles will help provide valuable feedback on the user interface and workflows during development as part of formative studies which are required for novel devices. Evidence exists [44] to support the human-AI team during usability studies and provision of information to users on the scenarios in which AI/ML devices can underperform to avoid the human becoming reliant on the results of an assistive tool for judgement.
Labelling/User training	All regulatory requirements including information for the user and in some situations - the patient. EN 82304 and many AI/ML guidances and proposed legislation stress the importance of providing clear and essential information relevant to the model performance, characteristics of the data used to train and test the model, acceptable inputs, contraindications, limitations of the model, guidance on interpreting result, and clinical workflow integration of the model. All this information is critical to the user to support the proper use of the device and to build comfortability of users with the device.
**Clinical deployment**	
Monitoring and maintenance	Following release of the device to the field for clinical use, post-market surveillance and post-market performance follow-up activities are critical for monitoring device performance, use, safety and security in the field using real world performance data. Controls can be built into AI/ML devices, which enable manufacturers to collect data on the performance both enabling analysis of the performance against the claimed performance and to identify drift, overfitting, unintended bias or model degradation. The FDA discuss the use of SaMD pre-specifications and change protocols [45], where as part of the pre-market approval manufactures can defined anticipated modification to the device performance as it learns from use data in the field. The uptake of this is yet to be seen as the concept of device performance changing in the field may not align to pathology laboratory practices or user comfortability, as there are qualification processes the laboratory require before a device can be used for clinical practice. The question remains, do pathologists and other users trust automated devices? In terms of monitoring for safety and security, maintenance programmes also need to include controls for monitoring the status of SOUPs and any potential vulnerabilities with the SOUPs, environment that device is held in (cloud or networked).
Risk management	The outputs of post-market surveillance and post-market performance follow-up activities should be used to review the risk assessment, overall residual risk assessment and benefit-risk profile for the device to confirm its ongoing suitability from a safety, performance and acceptability against the state of the art. Using the real world data available on the use of the device enables manufacturers to affirm or update their risk management file, and possibly intended purpose and design of the device. Given the novel nature of AI/ML medical devices and increase in DP devices being used in the field, more data will be available on similar devices to highlight any unforeseen or inappropriately addressed aspects of the risk management file.

Early definition of the intended purpose and performance goals for the AI/ML device is essential to ensure the data used to develop the model is appropriate, identify any potential bias in the dataset or model development methods and subsequent downstream design and development activities are appropriately planned for. The intended purpose should clearly define the intended use of the device: what is being detected and/or measured; the device function (screening, diagnosis, aid to diagnosis, prediction, companion diagnostic etc.); sample type; input information (giving information on the format, scanner type, staining etc.); intended patient population—taking into account variations in age, gender, race or other genetic factors; the intended user.

The target performance goals for the device should be defined before any model training is initiated, this should define against which clinically accepted state-of-the-art/gold standard the device is being assessed against, for example, manual assessment, molecular test or that no gold standard exists; what concordance is expected; and the number of samples required for training, testing and validation to provide confidence in the model performance.

Defining this information early enables clear scoping of the data required to train, test and validate the model, and sample type and format for subsequent clinical performance studies.

Depending on the number of samples available, and the sample size requirements for the performance goals, the samples shall be divided into three groups for training, testing and validation. The validation set must be different from the training and testing sets to ensure the model is being challenged by blind samples.

Data preparation requires processes for annotations, labelling, pre-processing and machine learning operations. Procedures for creating annotations should be prepared with suitably competent pathologists/clinical scientists. Where cell structures outside of the currently accepted clinical practice are being included, available literature should be reviewed and made available to those involved in the annotation process to ensure the pertinent information that will be used for model training have been included. Also key to the annotations process is definition of the quality acceptance criteria for samples during the annotation stages, clear guidance is essential of what is acceptable and where metadata is available - what is acceptable to ensure the samples sent forward for training align with the intended purpose for the device. These processes need to also include methods for issue management and investigation of samples which were initially sent for model training. One problem which is particularly challenging in pathology is often the lack of a definitive and objective ground truth. Since the annotation or labelling is being done manually by a pathologist, and knowing that for many tasks there is significant inter-pathologist variability in practice, this requires annotation protocols to account for this, with review by multiple pathologists and/or consensus ground truths being used as a proxy for an absolute ground truth.

Procedures for pre-processing need to take into account the format of the raw data (both image and metadata) and what format the model requires for learning, risks associated with the data conversion, the machine learning methods, the environment the sample data held in.

Machine learning operations should be prepared with suitably competent pathologists/clinical scientists for the aspects relating to feature selection and labelling, it is imperative that a rationale for feature selection and dependencies of features is defined to support downstream error analysis and explainability of the model performance. Other critical aspects to consider for the ML operations preparation are definition and qualification of tools for model training and annotations, definition of the model pipeline, error analysis methods and acceptance in the ML pipeline and model evaluation milestones, methods and metrics.

The output of the design and development activities will include the algorithm, software to support its use and environment, and also much include information for the user in the form of labelling and training materials. To ensure greater transparency for the user, these must include sufficient information about the intended purpose, any prerequisites for using the device, how the device works, performance claims and supporting information, the device limitations and, importantly, how the user should interpret the results of the device.

### Performance evaluation

Performance evaluation must be performed to collect the clinical evidence which proves that the device is safe, effective and meets the currently accepted state of the art. The types of studies are dependent on the device intended purpose, and peer-reviewed literature is a useful starting point to understand how other studies have been conducted [39]. The clinical performance goal should be statistically comparable to the state of the art with respect to diagnostic sensitivity, diagnostic specificity, positive predictive value, negative predictive value, likelihood ratio, expected values in normal and affected populations, as applicable. As with annotation, the evaluation must also account for the known problems of inter-pathologist variation, and the often limited availability of a definitive ground truth. Generally, for AI/ML devices a clinical performance study will be required, as it is a novel technology, and they can be performed on blind left-over samples or samples collected as part of a prospective study. These samples must not have been part of the AI model training or testing cohort. ISO 20916 provides details of how a clinical performance study should be conducted. There are many Medical Device Coordination Groups (MDCGs) (MDCG 2020-1, 2022-2), IMDRF (GHTF/SG5/N7 and /N8) and MedTech Europe guidances [40] available for guidance on performance evaluation studies.

### Regulatory approval

The regulatory approval route is dependent on the risk classification of the device, which is driven by the intended purpose. As AI/ML devices are likely higher risk, regulatory approval before release to market will be required. Each jurisdiction has different requirements for market access, examples are EU, UK, USA.

The authors strongly recommend engaging with the regulatory body, provide them information about the device they will be reviewing, establish the technical documentation in a manner that makes it easy for an external body to review and understand. Above all, have patience with the review body as AI/ML devices are new to them and they are learning too.

## Conclusion and future direction

For years now, DP and associated image analysis has been fundamental in the cancer tissue biomarker research strategy, an area that has been clearly enhanced by the application of AI tools as part of the image analysis approach. As such, DP/AI have become fundamental in the multi-modal analysis of cancer. While the number of DP/AI tools already available for diagnostic purposes is not small (see Fig. 3 above), the adoption of these tools by the pathology community has not been significant, indicating that a further improvement and evolution in the delivery of these tools in needed. Eventually, the application of new tissue-based algorithms, together with other tools able to extract further complexity from tissue hybridization-based experiments, has the potential of creating a new generation of “complex tissue biomarkers”. With awareness of the regulatory approval process, cancer scientists will have the opportunity of translating this complexity to efficient tools for patient diagnosis and therapeutic stratification.

In parallel to making the most of the complex content captured in images, the computational result of this analysis may need to face a broader degree of integration. AI/ML is percolating to other areas of medical information, such as radiology, laboratory medicine or simply to electronic patient records. The multi-modal analysis of these algorithmic outputs with computer science methods [41] may have a transformational effect in the way we practice cancer diagnostics. A robust image analysis approach to tissue pathology, as reviewed here, will be an essential pilar of such integration.
